# Research on the influence path of the unit environment of medical and health institutions on achievement transformation output

**DOI:** 10.1371/journal.pone.0295446

**Published:** 2023-12-14

**Authors:** Yanchao GAO, Hua GU

**Affiliations:** Hangzhou Institute of Medicine Chinse Academy of Sciences, Hangzhou, Zhejiang, China; Nanjing Audit University, CHINA

## Abstract

**Objective:**

To analyze the influence path of the interaction between the unit environment, achievement transformation willingness, and achievement transformation cognition on achievement transformation output to provide a basis for optimizing the achievement transformation environment of medical and health institutions and improving the efficiency of scientific and technological achievements transformation.

**Methods:**

Through the questionnaire survey, 292 data points were obtained. SPSS20.0 was used to conduct cross-table chi-square analysis and binary logistic regression analysis on the willingness, cognition, and output of scientific and technological achievements transformation. The process 14.0 plug-in is used to analyze the mediating effect of transformation cognition and the moderating effect of the unit environment.

**Results:**

Achievement transformation willingness has a significant positive impact on achievement transformation cognition and achievement transformation output. Achievement transformation cognition has a positive impact on achievement transformation output, and the mediating effect of transformation cognition is significant and partial. The unit environment has a negative moderating effect on the influence of achievement transformation willingness on achievement transformation cognition, and a positive moderating effect on the influence of achievement transformation willingness on achievement transformation output.

**Conclusion:**

Personal factors and unit achievement transformation environment have an obvious influence on the willingness and cognition of achievement transformation. It is necessary to optimize the environment of unit scientific and technological achievements transformation, improve the policy system of benefiting from the achievements of scientific and technological achievements transformation of medical and health personnel, implement the disposal right, income right and distribution right of scientific and technological achievements transformation of medical and health institutions, and stimulate the enthusiasm of scientific and technological achievements transformation of medical and health personnel.

## Introduction

**Fig 1:**
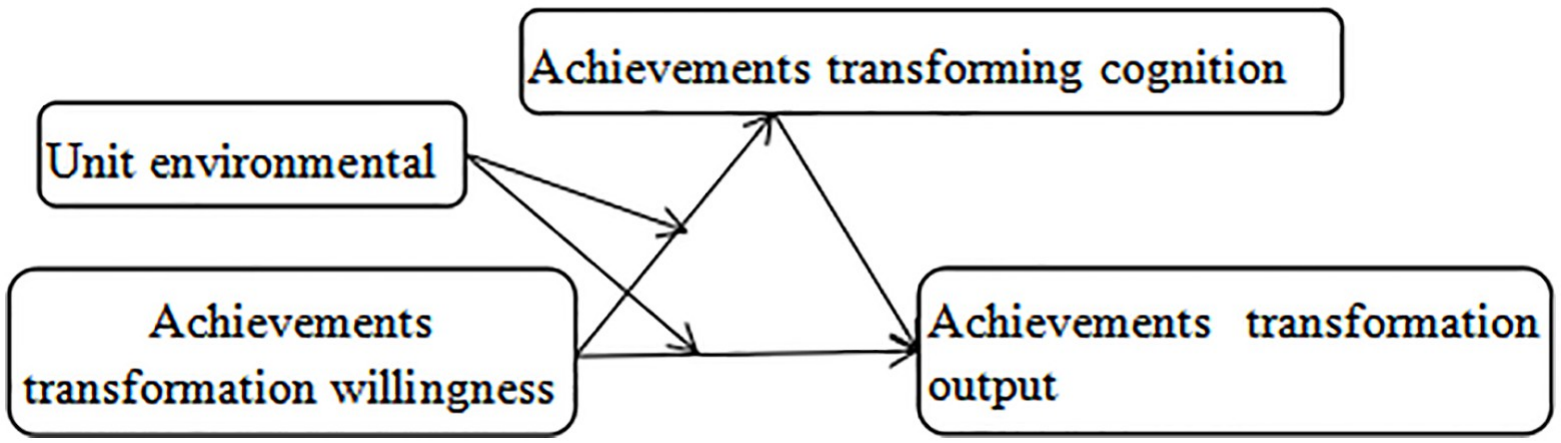
Theoretical model diagram.

Scientific and technological innovation is the core driving force for high-quality development. As an important carrier of scientific and technological innovation, medical and health institutions have outstanding characteristics of being talent-intensive, knowledge-intensive, and innovation-intensive [1]. In 2018, the performance appraisal of tertiary public hospitals in China amounted to 1.3593 million yuan per 100 health technicians [2], the conversion rate of scientific and technological achievements in medical and health institutions is only 5–10 per cent, compared with about 40 per cent in developed countries [3]. The low rate of transformation of scientific and technological achievements has become the most prominent problem restricting scientific and technological innovation in medical and health institutions [4]. The reasons for the low rate of transformation of scientific and technological achievements in medical and health institutions can be divided into internal incentive motivation and external incentive motivation [5, 6]. Internal incentive motivation is mainly manifested in the low willingness of medical and health personnel to transform scientific and technological achievements. The outstanding performance is that the enthusiasm of medical and health personnel is not high due to the lack of corresponding service support and the long transformation process [7]. Second, the cognition of the transformation of scientific and technological achievements is not high. Scientific and technological achievements are only used for performance appraisal, completion of subject tasks, and promotion of professional titles. Cognition of the policies, processes, and the importance of the transformation of scientific and technological achievements is not high [8, 9]. The external incentives are mainly manifested in the promotion and hindrance of the transformation environment of scientific and technological achievements of the unit to the transformation of scientific and technological achievements of medical and health personnel. The unit can improve the enthusiasm for the transformation of scientific and technological achievements of medical and health personnel by developing special policies and incentive systems for the transformation of scientific and technological achievements. On the contrary, the unit ignores the transformation of scientific and technological achievements, and the lack of specialized institutions to manage related affairs will weaken the enthusiasm of medical and health personnel to a certain extent [10, 11]. At present, research on the unit environment output on medical and health personnel outcome transformation concentrates on analysing the unit environment facilitating or hindering effect on medical and health personnel, but there is insufficient research on its influencing paths, and its interactions with other factors. This study analyzes the indirect influence of the unit environment on the transformation and output of scientific and technological achievements by studying the interaction between the unit environment and the willingness and cognition of achievement transformation. Therefore, we propose the following research design:

Theoretical model diagram (Fig 1). Transformation willingness refers to the strong degree of willingness to transform scientific and technological achievements, transformation cognition refers to the degree of awareness of the transformation of scientific and technological achievements, and transformation output refers to the results obtained from the transformation of achievements. According to existing research results, conversion output is affected by results conversion willingness and results conversion cognition. Results conversion cognition is affected by results conversion willingness; however, it is not known whether results conversion willingness can affect results conversion output by affecting results conversion cognition. Second, it is not known for the unit environment and results conversion willingness to interact with the results of the result conversion cognition and results conversion output. Therefore, we propose the following theoretical model and hypotheses:

**Hypothesis 1(H1)**: Achievement transformation willingness has a positive and significant impact on achievement transformation output.
**Hypothesis 2(H2)**: Achievement transformation cognition has a positive and significant impact on achievement transformation output.
**Hypothesis 3(H3)**: Achievement transformation willingness has a positive and significant impact on achievement transformation cognition. The influence of achievement transformation willingness on achievement transformation cognition is regulated by the unit environment.
**Hypothesis 4(H4)**: The unit environment has a moderating effect on the influence of achievement transformation willingness on achievement transformation cognition. The influence of achievement transformation willingness on achievement transformation output is regulated by the unit environment.
**Hypothesis 5(H5)**: The unit environment has a moderating effect on the influence of achievement transformation willingness on achievement transformation output. achievement transformation cognition mediates achievement transformation willingness.
**Hypothesis 6(H6)**: achievement transformation cognition mediates achievement transformation willingness and achievement transformation output.

## Materials and methods

An online questionnaire survey was conducted through the healthcare staff attending the course and 292 questionnaires were collected. The questionnaire includes: province, level of unit, type of unit, gender, age, title, years of working experience, education, type of title, environment of the unit, willingness cognition, and output of achievement transformation. Among them, 291 were from Zhejiang Province and 1 from Heilongjiang Province. Some options were deleted because they were missing, and 291 valid samples were retained. There were 97 males (33.3%) and 194 females (66.7%). There were 21 people aged 20–29 (7.2%), 149 people aged 30–39 (51.2%), 97 people aged 40–49 (33.3%), and 24 people aged 50–59 (8.2%). There were 6 people with ungraded titles (2.1%), 28 people with primary titles (9.6%), 127 people with intermediate titles (43.6%), 97 people with deputy senior titles (33.3%), and 33 people with senior titles (11.3%). There were 16 doctors (5.5%), 117 masters (40.2%), 155 undergraduates (53.3%), and 3 junior college and below (1%). There were 19 medical and health personnel at the provincial level (6.5%), 168 at the municipal level (57.7%), and 104 at the county level (35.7%). There were 280 people from medical and health institutions (96.2%), 3 people from scientific research institutes (1%), 1 person from higher medical colleges (0.3%), 3 people from disease control and prevention (1%), and 4 others (1.4%). Among the types of professional titles,12 were researchers (4.1%), 213 were medical technicians (73.2%), 18 were management technicians (6.2%), and 48 were others (16.5%). On the whole, the survey samples are mainly female, concentrated in the age group of 30–49 years old, with bachelor degree or above, intermediate title or above, medical technology, medical and health institutions, in line with the distribution law of general medical and health personnel.

Excel is used to process, summarize, and clean the data, and *SPSS20*.*0* and the *Process* are used to verify and process the model. Descriptive statistical analysis, cross-table chi-square analysis, binary logistic regression analysis, and mediating and moderating effects were performed. P < 0.05 indicated that the difference was statistically significant.

### Statistical analysis

#### Demographic chi-square analysis

In the unit environment, achievement transformation cognition, and achievement transformation output demographic chi-square analysis (Table 1), it can be seen that the unit type, unit level, and professional title have obvious tendencies in the results of the cross-table chi-square analysis of achievement transformation output. With an increase in unit and professional title level, the proportion of achievement transformation output of respondents is higher. Regarding the type of professional title, the probability of achievement transformation and output of researchers and medical technology is significantly higher than that of the management.

**Table 1:** Demographic chi-square analysis of the achievement transformation output.

Achievements transformation output			*χ* ^2^	Achievements transformation output			*χ* ^2^
	No	Yes			No	Yes	
**Unit type**			**9.688** *	**Title**			**4.807** *
Medical and health institutions	165	116		Ungraded	4	2	
Research institutes	0	3		Primary	23	6	
Medical colleges and universities	1	0		medium-grade professional title	75	52	
Disease control	3	0		Deputy high title	52	45	
Other	2	2		title of a senior professional post	17	16	
**Unit level**			**15.269** **				
Provincial level	4	15					
Municipal level	96	73					
County level	71	33					

* means P < 0.05,

** means P < 0.01.

The chi-square analysis of the willingness and cognition of achievements transformation (Table 2) shows that there is a clear tendency of sex in the transform achievements willingness, and the enthusiasm of women ’s willingness to transform achievements is higher than that of men (Women physicians are under stronger pressure to be promoted). The level of education has a clear tendency in achievement transformation cognition, and master ’s degree personnel have a higher degree of cognition (Physicians with doctoral degrees have a heavier workload in the daily work).

**Table 2:** Demographic chi-square analysis of willingness and cognition of achievement transformation.

Achievement transformation Willingness			*χ* ^2^		Achievement transformation cognition			*χ* ^2^
	No	Yes			incomprehension	general	acquaint	
**Gender**			5.944*	**Education level**				**8.393** *
Male	18	79		Doctor	6	9	1	
Female	17	178		Masters	21	68	29	
				Undergraduate	24	76	55	
				Junior college and below	0	2	1	

* means P < 0.05,

** means P < 0.01.

#### Analysis of the mediating effect of achievement transformation cognition

Analysis of the intermediary effect of achievement transformation cognition Table 3, divided into two steps to verify the intermediary effect of achievement transformation cognition on achievement transformation willingness. First verify the effect of independent variables on intermediary variables, and then verify the effect of independent variables and intermediary variables on dependent variables. Both are significant, which means that the intermediary effect exists. First, transform achievements willingness impacts on achievement transformation cognition, with an explanation degree of 4.9%. The model passed the significance test, and the coefficient of transformation achievements willingness is 0.46, P < 0.05,95% confidence interval does not contain 0, that is, transformation achievements willingness has a positive and significant impact on achievement transformation cognition. Second, the influence of cognition and willingness of achievement transformation on achievement transformation output. Because achievement transformation output is a nominal variable (0 = none, 1 = yes), the-2 log likelihood value is 392.92, and the CoxSnell R-sq is 0.042, the model passes the significance test. The influence coefficient of achievement transformation willingness on achievement transformation output (β = 0.374, p < 0.05), that is, the stronger the achievement transformation willingness, the higher the probability of achievement transformation output. The influence coefficient of achievement transformation cognition on achievement transformation output (β = 1.002, p < 0.05), that is, the higher achievement transformation cognition, the higher the probability of achievement transformation output. That is to say, it supports hypothesis H1: achievement transformation willingness has a positive and significant impact on achievement transformation output; support hypothesis H2: achievement transformation cognition has a positive and significant impact on achievement transformation output; support hypothesis H3: achievement transformation willingness has a positive and significant impact on achievement transformation cognition.

**Table 3:** Regression analysis of the mediating effect of achievement transformation cognition.

independent variable	coeff	se	t	p	LLCI	ULCI
constant	1.7143	.1115	15.3701	.0000	1.4948	1.9338
achievement transformation willingness	.4608	.1189	3.8761	.0001	.2268	.6948
dependent variable: Achievement transformation cognition; R = 0.2219, R-sq = 0.0493, F = 15.024, P<0.05						
independent variable	coeff	se	t	p	LLCI	ULCI
constant	-2.0456	.5422	-3.7729	.0002	-3.1083	-.9829
achievement transformation willingness	.3740	.1861	2.0094	.0445	.0092	.7389
Achievement transformation cognition	1.0017	.4489	2.2316	.0256	.1219	1.8816
dependent variable: achievements transformation output; -2LL = 392.92, CoxSnell = 0.042, Model LL = 12.273, P<0.05						

Table 4 shows the direct effect of achievement transformation willingness on achievement transformation output was 1.002, p < 0.05,95% confidence interval excludes 0. The indirect effect of achievement transformation willingness indirectly affects achievement transformation output by influencing achievement transformation cognition. The effect value is 0.1724, and the 95% confidence interval does not contain 0, that is, the achievement transformation cognition intermediary effect exists and is a partial intermediary. Support Hypothesis H6: achievement transformation cognition has a significant mediating effect on achievement transformation willingness and output.

**Table 4:** Analysis of the mediating effect value of achievement transformation cognition.

Direct effect of: Willingness of achievement transformation→Achievements transformation output					
Effect	SE	Z	p	LLCI	ULCI
1.0017	.4489	2.2316	.0256	.1219	1.8816
Indirect effect of: achievement transformation willingness→Achievement transformation cognition→Achievements transformation output					
	Effect	Boot SE		BootLLCI	BootULCI
Achievement transformation cognition	.1724	.1020		.0208	.4335

#### Analysis of the moderating effect of the unit environment on the willingness and cognition of achievement transformation

Table 5 shows the unit environment has a positive and significant impact on achievement transformation cognition (β = 1.06, p < 0.05), and the unit environment has a positive and significant impact on achievement transformation cognition (β = 0.334, p < 0.05), that is, the better the unit transformation environment, the more profound the respondents’ cognition of achievement transformation. The unit environment has a negative moderating effect on achievement transformation willingness and the cognitive regulation effect of achievement transformation (β = -0.321, p < 0.05), that is, the unit environment has a negative moderating effect on achievement transformation willingness, indicating that when the respondents’ willingness of achievement transformation is high; and the unit environment is poor, their cognition of achievement transformation will be reduced.

**Table 5:** Analysis of the moderating effect of the unit environment on achievement transformation willingness on achievement transformation cognition.

independent variable	coeff	se	t	p	LLCI	ULCI
constant	1.0850	.2776	3.9077	.0001	.5385	1.6314
achievement transformation willingness	1.0637	.2960	3.5935	.0004	.4811	1.6463
unit environmental	.3337	.1350	2.4718	.0140	.0680	.5995
moderating effect	-.3207	.1428	-2.2467	.0254	-.6017	-.0398
dependent variable: achievement transformation cognition R = 0.2632, R-sq = 0.0693, F = 7.143, P<0.05						

**Fig 2:**
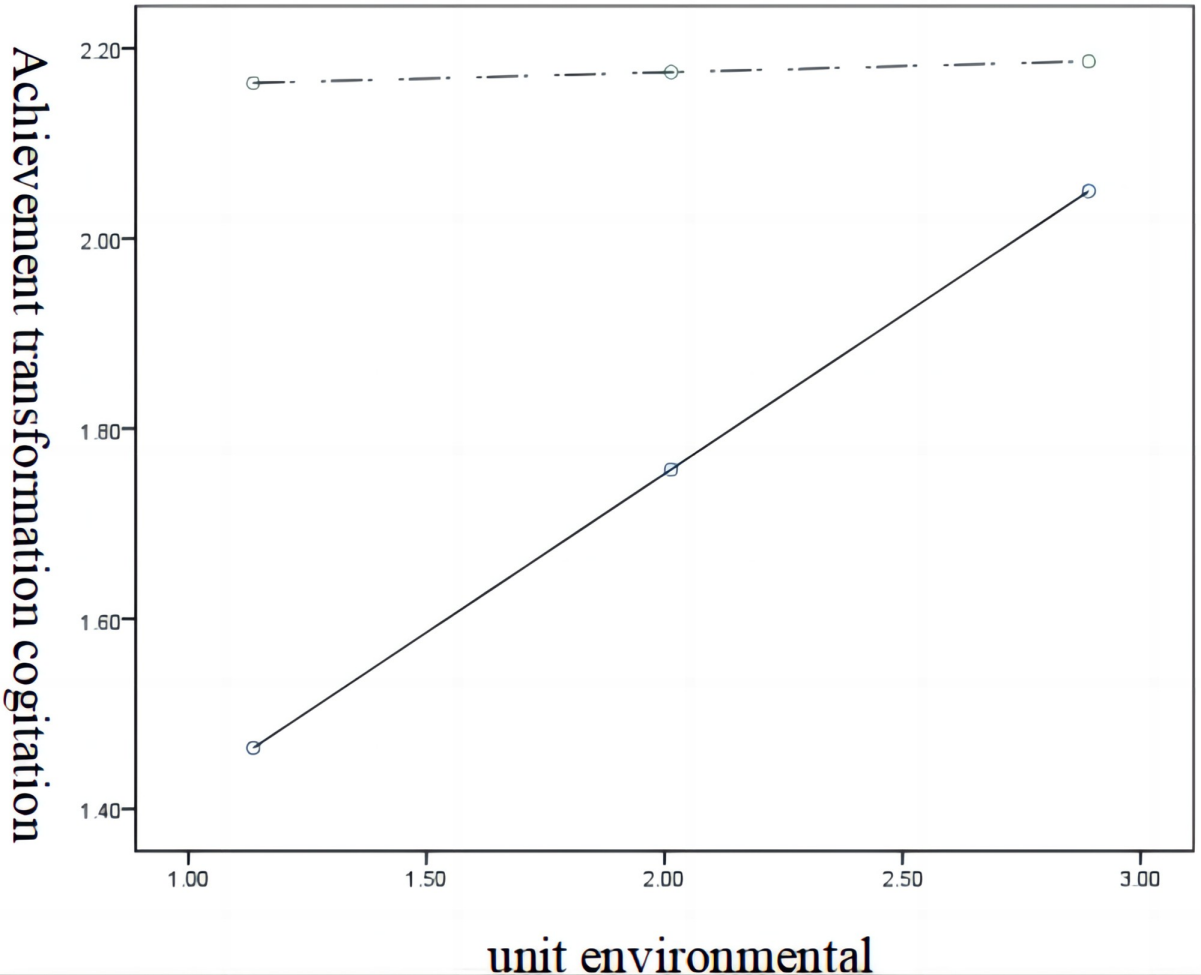
Schematic diagram of the moderating effect of the unit environment on achievement transformation willingness and cognition.

Fig 2 shows the effect of the unit environment on achievement transformation willingness and the regulation effect of achievement transformation cognition. It can be seen that under the action of the unit environment, the straight line of the effect value of achievement transformation willingness at high and low levels (M ± SD) is non-parallel, that is, there is a regulatory effect.

The highest-order unconditional moderating effect test (Table 6), in which the moderating effect increases the interpretation of achievement transformation willingness to achievement transformation cognition by 1.6%. The significance test, supports hypothesis H4: the unit environment has a negative moderating effect on the achievement transformation willingness and cognition.

**Table 6:** The highest order unconditional adjustment effect test.

	R2-chng	F	df1	df2	p
X*W	.0163	5.0476	1.0000	288.0000	.0254

#### Analysis of the adjustment effect of the unit environment on the willingness and output of achievement transformation

The adjustment effect of the unit environment on achievement transformation willingness on achievement transformation output (Table 7). Achievement transformation cognition has a positive and significant impact on achievement transformation output (β = 0.438, p < 0.05), achievement transformation willingness has a negative and significant impact on achievement transformation output (β = -2.27, p < 0.05), and the unit environment has a positive impact on achievement transformation cognition but not significant (β = -0.469, p > 0.05). The unit environment has a positive adjustment effect on achievement transformation willingness and achievement transformation output (β = 1.615, p < 0.05), that is, the unit environment has a positive adjustment effect on achievement transformation willingness, indicating that when the unit transformation environment is better, the higher the willingness of the respondents to transform the results, the higher the probability of the respondents to transform the results.

**Table 7:** Analysis of the adjustment effect of the unit environment on the willingness and output of achievement transformation.

	coeff	se	Z	p	LLCI	ULCI
constant	-1.2948	1.054	-1.2283	.2193	-3.3609	.7712
achievement transformation cogitation	.4383	.2069	2.1181	.0342	.0327	.8439
achievement transformation willingness	-2.273	1.1196	-2.0302	.0423	-4.4673	-.0786
unit environmental	-.4686	.5339	-.8776	.3801	-1.5150	.5779
moderating effect	1.6147	.5614	2.8762	.0040	.5144	2.7151
dependent variable: Achievements transformation output-2LL = 328.699, CoxSnell = 0.2064, Model LL = 67.496, P<0.05						

**Fig 3:**
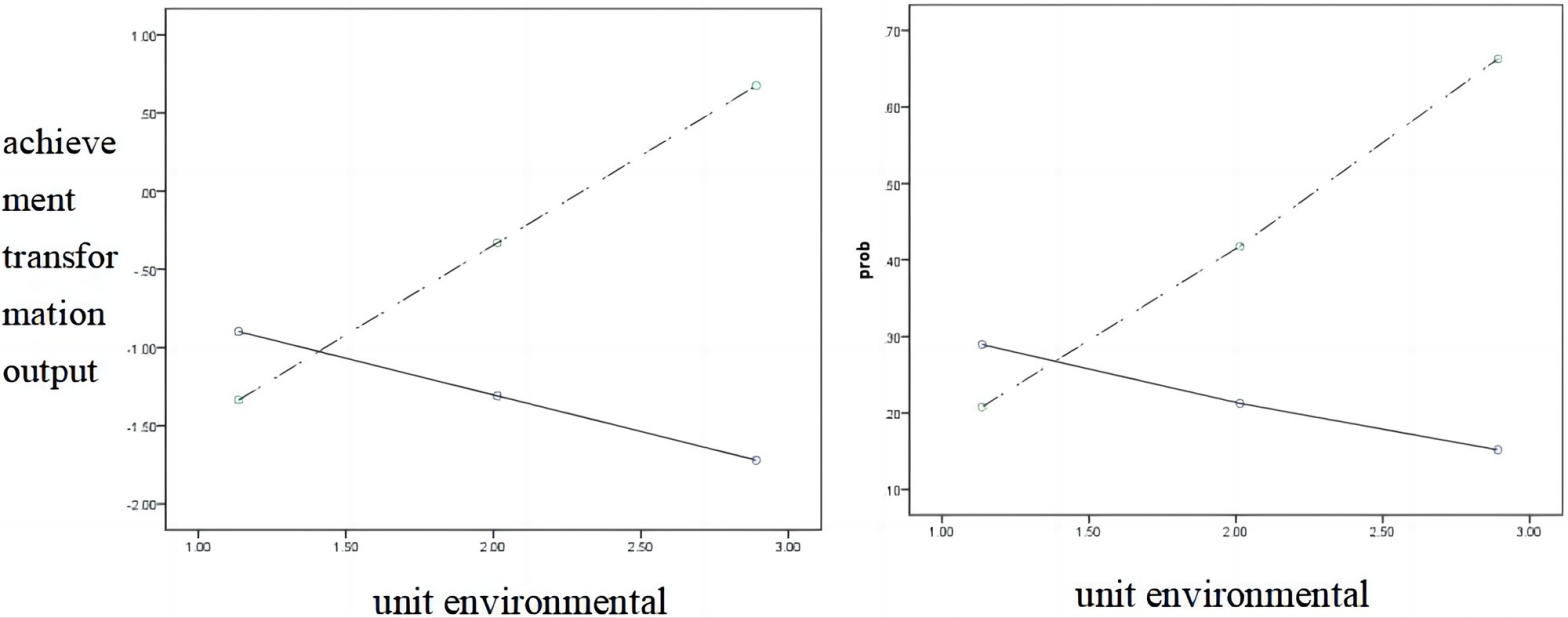
Two-condition adjustment effect diagram of achievement transformation output (left achievement transformation output = 0; right outcome transformation output = 1).

Fig 3 shows the effect of the unit environment on achievement transformation willingness and the adjustment effect of achievement transformation output. It can be seen that under the action of the unit environment, the straight line of the effect value of achievement transformation willingness at high and low levels (M ± SD) is non-parallel, that is, there is an adjustment effect.

According to the numerical table of the unit environment adjustment effect (Table 8), under a high level of the unit environment, the 95 confidence interval of achievement transformation willingness for achievement transformation output adjustment effect does not contain 0, which is significant. The indirect effect was significant at the middle and low levels. The willingness to transform the results affects the output effect of the results by affecting their cognition. That is to say, it supports hypothesis H5: the unit environment has a positive moderating effect on achievement transformation willingness and achievement transformation output, and the moderating mediating effect is significant (χ2 = 8.58, p < 0.05).

**Table 8:** Numerical table of the unit environmental regulation effect.

Direct effect with regulation (Willingness of achievement transformation- > Achievements transformation output)						
unit environmental	Effect	se	Z	p	LLCI	ULCI
1.1364	-0.4379	0.5996	-0.7304	0.4652	-1.6130	0.7372
2.0137	0.9786	0.4756	2.0578	0.0396	0.0465	1.9107
2.8910	2.3951	0.7602	3.1506	0.0016	0.9051	3.8851
With moderating indirect effect (achievement transformation willingness- > Achievement transformation cognition- > Achievements transformation output)						
unit environmental	Effect	BootSE			BootLLCI	BootULCI
1.1364	0.3065	0.1650			0.0336	0.6758
2.0137	0.1832	0.1037			0.0181	0.4210
2.8910	0.0598	0.0905			-0.0872	0.2742
X*W: Chi-sq = 8.5833, df = 1, p = 0.0034						

## Results and discussion

### Individual factors have different effects on the willingness, cognition, and output of achievement transformation

Men are more willing to transform their achievements in terms of gender, which is consistent with the existing research [12]. The willingness of medical and health personnel with innovative ideas to transform achievements is significantly higher than that without innovative ideas; that is, the more innovative ideas medical and health personnel have, the more willing they are to transform scientific and technological achievements. The higher the level of education, the higher level of transformation cognition of scientific and technological achievements, the higher the overall male achievement transformation cognition [13], the higher achievement transformation cognition of the respondents who are more satisfied with the achievement transformation of the unit, the higher the cognition of the achievement transformation of the younger than the older [14], the higher the degree of attention paid by the unit to the transformation of scientific and technological achievements, the higher the cognition of the transformation of achievements, and the higher the cognition of the transformation of achievements. In other words, the willingness and cognition of achievement transformation are obviously affected by personal factors. Since men have higher demands for title and position promotion than women, and the transformation of scientific and technological achievements weighs more and more in the evaluation, the willingness and cognition of transformation of scientific and technological achievements are relatively higher among men. Second, the attitude of the unit towards the transformation of scientific and technological achievements; and the management level of the transformation of scientific and technological achievements can obviously guide the willingness and cognition of the medical and health personnel. Such as China’s assessment of tertiary public hospitals to increase the assessment of scientific and technological achievements to increase the proportion of the assessment of the transformation of scientific and technological achievements, obviously leading hospitals to pay attention to the transformation of scientific and technological achievements, which in turn leads medical and health care personnel to pay attention to scientific and technological achievements.

### Relationship between achievement transformation willingness, achievement transformation cognition, and achievement transformation output

Achievement transformation willingness has a positive and significant impact on achievement transformation cognition and achievement transformation output. achievement transformation cognition has a positive and significant impact on achievement transformation output. achievement transformation cognition has a significant mediating effect on achievement transformation willingness and achievement transformation. This shows that in the chain of scientific and technological achievements transformation, the willingness to transform achievements is the starting point of achievement transformation, and the willingness to transform achievements further deepens the understanding of achievement transformation and brings about the output of achievements. At the same time, achievement transformation willingness can directly affect achievement transformation output; that is, the intensity of achievement transformation willingness is one of the factors affecting achievement transformation output. Achievement transformation willingness and cognition are important factors influencing outcome outputs, which is consistent with existing research and clarifies the pathways of outcome output impacts.

### Moderating effect of the unit environment

The regulatory role of the unit environment is manifested in two main aspects. First, the unit achievement transformation environment regulates the impact of the willingness to transform achievements on achievement transformation cognition, that is, the willingness to transform achievements is limited by the unit achievement transformation environment. When the unit achievement transformation environment is poor (If the unit has formulated policies related to the transformation of achievements, set up a responsible organisation, and has corresponding full-time staff, we believe that the unit’s environment for the transformation of achievements is excellent, and vice versa is poor), the individual’s willingness to transform achievements is inhibited, and the cognitive level of achievement transformation is reduced. Second, the unit environment has a positive moderating effect on achievement transformation willingness and achievement transformation output. When the environment of unit achievement transformation is better, the higher achievement transformation willingness, the greater the probability of achievement transformation output. It shows that a good achievement transformation environment of the unit is beneficial to improve the enthusiasm of medical and health personnel in the transformation of scientific and technological achievements, and at the same time, when the unit environment is poor, it will hinder achievement transformation cognition. The unit environment affects the output of results by influencing the willingness and cognition of individuals. Here we clarify the path of influence of the external environment on promoting the transformation of scientific and technological achievements, which complements the existing research.

## Conclusions

Therefore, to promote the transformation of scientific and technological achievements, we need to make efforts from both inside and outside, on the one hand, to guide individuals on the transformation of achievements enthusiasm, and constantly deepen the knowledge of the transformation of achievements. Second, we should improve the system and organisational construction of the unit environment, guide individuals to invest in the transformation of achievements through the system and organisational construction and strengthen the depth of individuals’ knowledge of the transformation of achievements through the popularisation of policies. To a certain extent, the unit environment is more important for achievement transformation. The following suggestions are put forward: First, optimize the construction of the environment for the transformation of scientific and technological achievements, express special policies and management methods to promote the transformation of scientific and technological achievements, improve the policy system for the benefit of medical and health personnel, and establish special management institutions and assessment methods for the transformation of scientific and technological achievements. The second to establish and improve the incentive mechanism and promotion methods for the transformation of scientific and technological achievements, incorporate the transformation of scientific and technological achievements into the evaluation criteria of professional titles, implement the disposal, income, and distribution rights of the transformation of scientific and technological achievements in medical and health institutions, and stimulate the enthusiasm of medical and health personnel for the transformation of scientific and technological achievements. The third is to increase the publicity and correct guidance of the transformation of scientific and technological achievements, increase the publicity of policies such as the transformation of scientific and technological achievements, strengthen the publicity of the transformation process of scientific and technological achievements, and improve the cognition of medical and health personnel regarding the transformation of scientific and technological achievements. Due to time and financial constraints, the study was inadequate in terms of the amount of data collected and the need for further research on data interrelationships. In the future, we will further explore the interactions between national and unit policies in terms of their impact on the transformation of individual achievements.

## Supporting information

S1 Data (SAV)
